# Gypenosides attenuate the development of L-DOPA-induced dyskinesia in 6-hydroxydopamine-lesioned rat model of Parkinson’s disease

**DOI:** 10.1186/s12868-015-0163-5

**Published:** 2015-04-21

**Authors:** Keon Sung Shin, Ting Ting Zhao, Keun Hong Park, Hyun Jin Park, Bang Yeon Hwang, Chong Kil Lee, Myung Koo Lee

**Affiliations:** College of Pharmacy and Research Center for Bioresource and Health, Chungbuk National University, 1, Chungdae-ro, Seowon-gu Cheongju, 362-763 Republic of Korea

**Keywords:** *Gynostemma pentaphyllum*, 6-Hydroxydopamine-lesioned rats, Dyskinesia, Body and locomotive AIMs scores, ∆FosB, ERK1/2, Adjuvant therapeutics

## Abstract

**Background:**

Gypenosides (GPS) and ethanol extract of *Gynostemma pentaphyllum* (GP-EX) show anxiolytic effects on affective disorders in 1-methyl-4-phenyl-1,2,3,6-tetrahydropyridine-lesioned mouse model of Parkinson’s disease (PD). Long-term administration of L-3,4-dihydroxyphenylalanine (L-DOPA) leads to the development of severe motor side effects such as L-DOPA-induced-dyskinesia (LID) in PD. The present study investigated the effects of GPS and GP-EX on LID in a 6-hydroxydopamine (6-OHDA)-lesioned rat model of PD.

**Results:**

Daily administration of L-DOPA (25 mg/kg) in the 6-OHDA-lesioned rat model of PD for 22 days induced expression of LID, which was determined by the body and locomotive AIMs scores and contralateral rotational behaviors. However, co-treatments of GPS (25 and 50 mg/kg) or GP-EX (50 mg/kg) with L-DOPA significantly attenuated the development of LID without compromising the anti-parkinsonian effects of L-DOPA. In addition, the increases in ∆FosB expression and ERK1/2 phosphorylation in 6-OHDA-lesioned rats induced by L-DOPA administration were significantly reduced by co-treatment with GPS (25 and 50 mg/kg) or GP-EX (50 mg/kg).

**Conclusion:**

These results suggest that GPS (25 and 50 mg/kg) and GP-EX (50 mg/kg) effectively attenuate the development of LID by modulating the biomarker activities of ∆FosB expression and ERK1/2 phosphorylation in the 6-OHDA-lesioned rat model of PD. GPS and GP-EX will be useful adjuvant therapeutics for LID in PD.

## Background

Parkinson’s disease (PD) is a progressive neurological disorder mainly due to the degeneration of dopaminergic neuronal cells in the substantia nigra pars compacta [1]. The precursor of dopamine, L-3,4-dihydroxyphenylalanine (L-DOPA), is the most effective known therapy for controlling the motor symptoms in PD such as slowness, rigidity, resting tremor, and postural instability [2,3]. However, chronic L-DOPA administration results in a loss of drug efficacy and irreversible adverse effects, and also leads to the development of sever motor fluctuations such as L-DOPA-induced-dyskinesia (LID) [4]. Typically, dyskinesia can occur in association with high concentrations of L-DOPA in the brain and maximum improvement in the motor responses [5,6]. The most common type of dyskinesia called peak-dose dyskinesia, occurs throughout the “on” time period to response of L-DOPA administration.

Although the mechanisms underlying the development and expression of LID are complex, the development of LID is associated with changes in the gene and protein expressions of ∆FosB and extracellular signal-regulated kinases (ERK1/2) in nigral dopamine cell loss [7,8]. LID increases striatal levels of ∆FosB and ERK1/2 [7,8]. Chronic L-DOPA administration in 6-hydroxydopamine (6-OHDA)-lesioned rats also induces FosB-like protein ∆FosB in the dopamine-denervated striatum that develops dyskinesias [9]. ∆FosB protein is induced by L-DOPA administration in the striatum of 6-OHDA-lesioned rats [8]. In addition, increased ERK1/2 phosphorylation by dopamine agonists in 6-OHDA-lesioned striatal neurons is a possible mechanism of LID [10,11]. ERK1/2 phosphorylation correlates with increased ∆FosB and dyskinesia in the depleted striatum of 6-OHDA-lesioned rats [8]. Therefore, ∆FosB expression and ERK1/2 phosphorylation in the striatum are implicated in the development of LID as biological molecular markers [8,9].

*Gynostemma pentaphyllum* Makino (Cucurbitaceae) (GP) is a well-known herbal medicinal plant in Southeast Asia. GP contains approximately 90 dammarane-type glycosides (*Gynostemma* total saponins, gypenosides; GPS), flavonoids, polysaccharides, amino acids, vitamins, and some essential elements [12]. Recently, GPS has been shown to have anxiolytic effects on affective disorders and neuroprotective effects in 1-methyl-4-phenyl-1,2,3,6-tetrahydropyridine (MPTP)-lesioned mouse model of PD [13,14]. In addition, ethanol extract from GP (GP-EX) protects the dopaminergic neurons in 6-OHDA-lesioned rat model of PD [15]. GP-EX also has anti-stress effects in mice [16].

Although GPS has therapeutic benefits for several neurodegenerative diseases including PD, the effects of GPS on the development of LID have not been identified yet. In this study, therefore, we examined the effects of GPS and GP-EX on LID using the rat model of LID. After treatments with GPS and GP-EX in L-DOPA-administered 6-OHDA-lesioned rats, LID was analyzed by using the score of abnormal involuntary movements (AIMs) (body AIMs score and locomotive AIM score), contralateral rotational behaviors, and the biomarker activities of ∆FosB expression and ERK1/2 phosphorylation.

## Results

### Effects of GPS and GP-EX on LID

**Figure 1:**
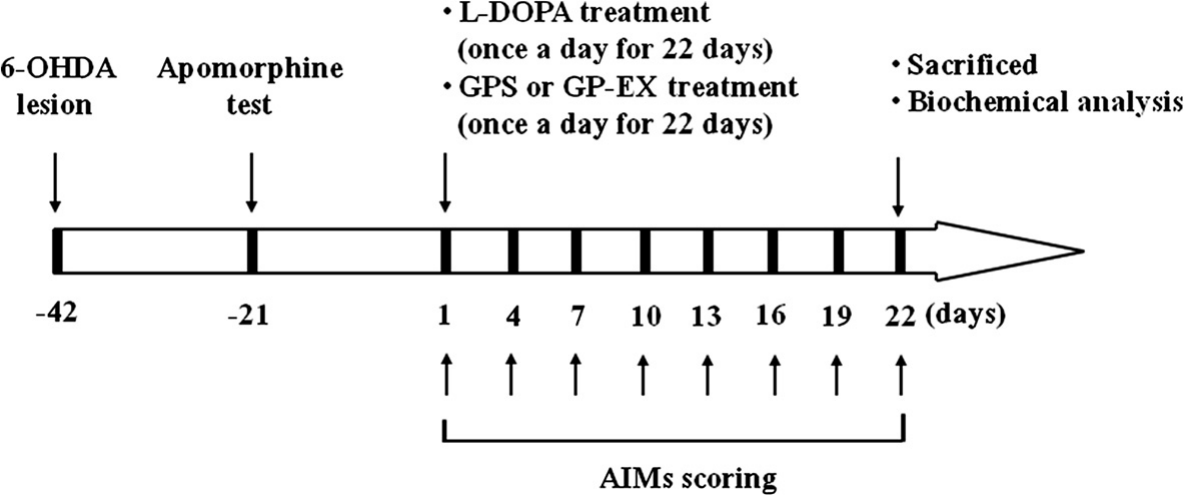
Experimental design. Rats (SD, male, 200–250 g) were divided into five groups [8–10 animals per each group: saline-treated group, L-DOPA-treated group, L-DOPA and GPS (25 or 50 mg/kg)-treated group, L-DOPA and GP-EX (50 mg/kg)-treated group] and PD models were established by 6-OHDA lesion (8 μg/2 μl). Three weeks after the 6-OHDA lesion, the apomorphine-induced rotational test was carried out to assess the efficacy of the 6-OHDA lesion. L-DOPA (25 mg/kg, i.p.) and benserazide (15 mg/kg, i.p.) treatment was started 6 weeks after the 6-OHDA lesion once a day for 22 days. Either GPS (25 and 50 mg/kg, p.o.) or GP-EX (50 mg/kg, p.o.) was given 30 min prior to L-DOPA treatment once a day for 22 days. The AIMs were scored for 1 min every 20 min for total 180 min after L-DOPA treatment on the indicated day. On day-22, after behavioral measurements, the animals were sacrificed for biochemical analyses.

**Figure 2:**
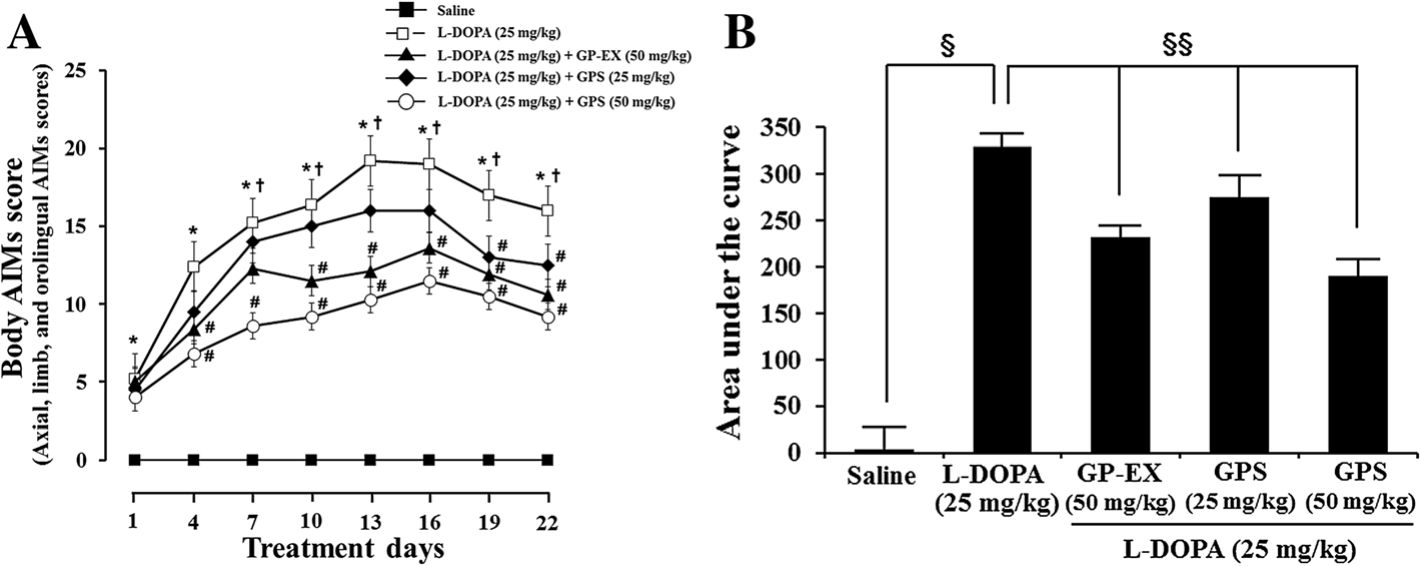
Effects of GPS and GP-EX on body AIMs score in 6-OHDA-lesioned rats. L-DOPA (25 mg/kg, i.p.) and benserazide (15 mg/kg, i.p.) treatment was started 6 weeks after the 6-OHDA lesion once a day for 22 days. Either GPS (25 and 50 mg/kg, p.o.) or GP-EX (50 mg/kg, p.o.) was given 30 min prior to L-DOPA treatment once a day for 22 days. The axial, limb, and orolingual AIMs were calculated by adding each of the individual dyskinesia scores as body AIMs. **A**: body AIMs; **B**: area under the curve of body AIMs. The results are expressed as mean ± S.E.M. for 8–10 animals/group. –■–, saline treatment; −□–, L-DOPA; −▲–, L-DOPA + GP-EX (50 mg/kg); −◆–, L-DOPA + GPS (25 mg/kg); −○–, L-DOPA + GPS (50 mg/kg). **A**: ^*^ *P* < 0.05 compared with saline-treated group at each treatment day; ^#^ *P* < 0.05 compared with L-DOPA alone-treated group at each treatment day (non-parametric Kruskal-Wallis one-way ANOVA test); ^†^ *P* < 0.05 compared with the score of day-1 (Friedman repeated measures ANOVA test), **B**: ^§^ *P* < 0.05 compared with saline-treated group; ^§§^ *P* < 0.05 compared with L-DOPA alone-treated group (non-parametric Kruskal-Wallis one-way ANOVA test).

In order to measure the effects of GPS and GP-EX on LID, the treatment schedule including the behavioral and biochemical analyses is presented in Figure 1. The body AIMs score (total AIMs score; the integrated axial, limb, and orolingual AIMs scores) and locomotive AIMs score using the different dyskinesia subtypes (axial, limb, orolingual, and locomotive AIMs score) were analyzed separately. The daily repeated administration of L-DOPA (25 mg/kg) induced an increase over time in the body AIMs score in 6-OHDA-lesioned rats. LID by the body AIMs score was observed at day-4 and continued to increase at day-10, reaching its maximum at day-13 and day-16 (Figure 2A). The body AIMs score of LID was slightly decreased at day-19 and day-22, which was comparable with the previously reported results [17]. In this study, the behavioral tests and biochemical analyses were examined for 22 days.

The body AIMs score and locomotive AIMs score were not affected in the sham-operated rats, which had unaffected AIMs scores. However, treatment with GPS (25 and 50 mg/kg) or GP-EX (50 mg/kg) in 6-OHDA-lesioned rats treated with L-DOPA for 22 days displayed much less severe dyskinesia (*P* < 0.05), compared with 6-OHDA-lesioned rats treated with L-DOPA (Figure 2A and B).

**Figure 3:**
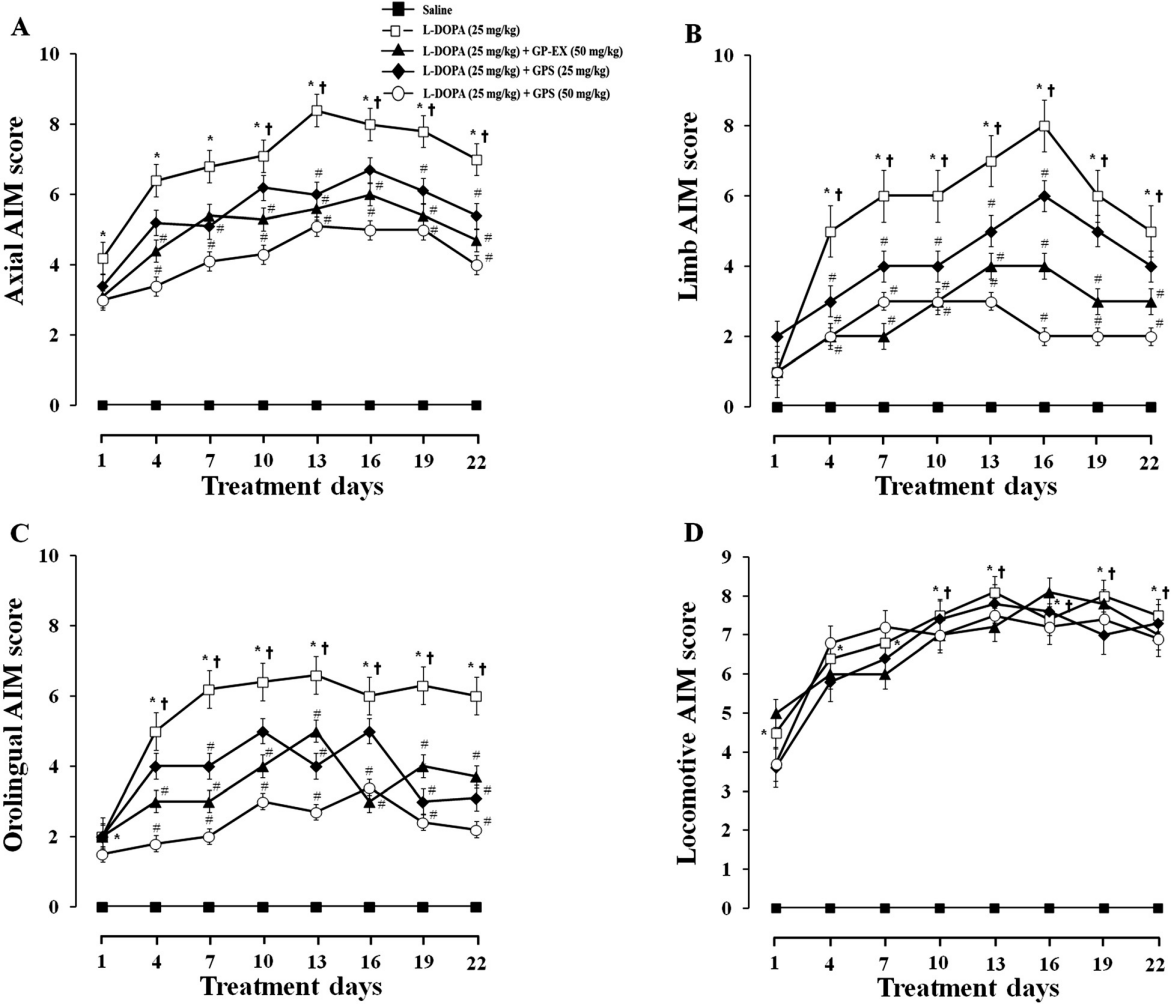
Effects of GPS and GP-EX on L-DOPA-induced AIMs in 6-OHDA-lesioned rats. L-DOPA (25 mg/kg, i.p.) and benserazide (15 mg/kg, i.p.) treatment was started 6 weeks after the 6-OHDA lesion once a day for 22 days. Either GPS (25 and 50 mg/kg, p.o.) or GP-EX (50 mg/kg, p.o.) was given 30 min prior to L-DOPA treatment once a day for 22 days. The AIMs were scored for 1 min every 20 min for total 180 min after L-DOPA treatment on the indicated day according to the description in the Materials and methods section. **A**: axial AIMS; **B**: limb AIMs; **C**: orolingual AIMs; **D**: locomotive AIMs. The results are expressed as mean ± S.E.M. for 8–10 animals/group. –■–, saline treatment; −□–, L-DOPA; −▲–, L-DOPA + GP-EX (50 mg/kg); −◆–, L-DOPA + GPS (25 mg/kg); −○–, L-DOPA + GPS (50 mg/kg). ^*^ *P* < 0.05 compared with saline-treated group at each treatment day; ^#^ *P* < 0.05 compared with L-DOPA alone-treated group at each treatment day (non-parametric Kruskal-Wallis one-way ANOVA test); ^†^ *P* < 0.05 compared with the score of day-1 (Friedman repeated measures ANOVA test).

Next, the effects of GPS and GP-EX on the different dyskinesia subtypes such as the body AIMs score (the axial, limb and orolingual AIMs score) and locomotive AIMs score were analyzed. The axial, limb and orolingual AIMs score was increased by the daily repeated administration of L-DOPA (25 mg/kg) for 22 days in 6-OHDA-lesioned rats (Figure 3A–D). In contrast, daily repeated treatments with GPS (25 and 50 mg/kg) or GP-EX (50 mg/kg) prior to L-DOPA administration in 6-OHDA-lesioned rats treated with L-DOPA significantly attenuated the development of L-DOPA-induced body AIMs (Figure 3A–C).

In addition, the locomotive AIMs score was not affected by repeated treatments with L-DOPA (25 mg/kg) in 6-OHDA-lesioned rats. Treatment with GPS (25 and 50 mg/kg) or GP-EX (50 mg/kg) in 6-OHDA-lesioned rats treated with L-DOPA also did not reduce the locomotive AIMs score, compared with GPS- or GP-EX-untreated group (Figure 3D).

### Effects of GPS and GP-EX on contralateral rotational behavior

**Figure 4:**
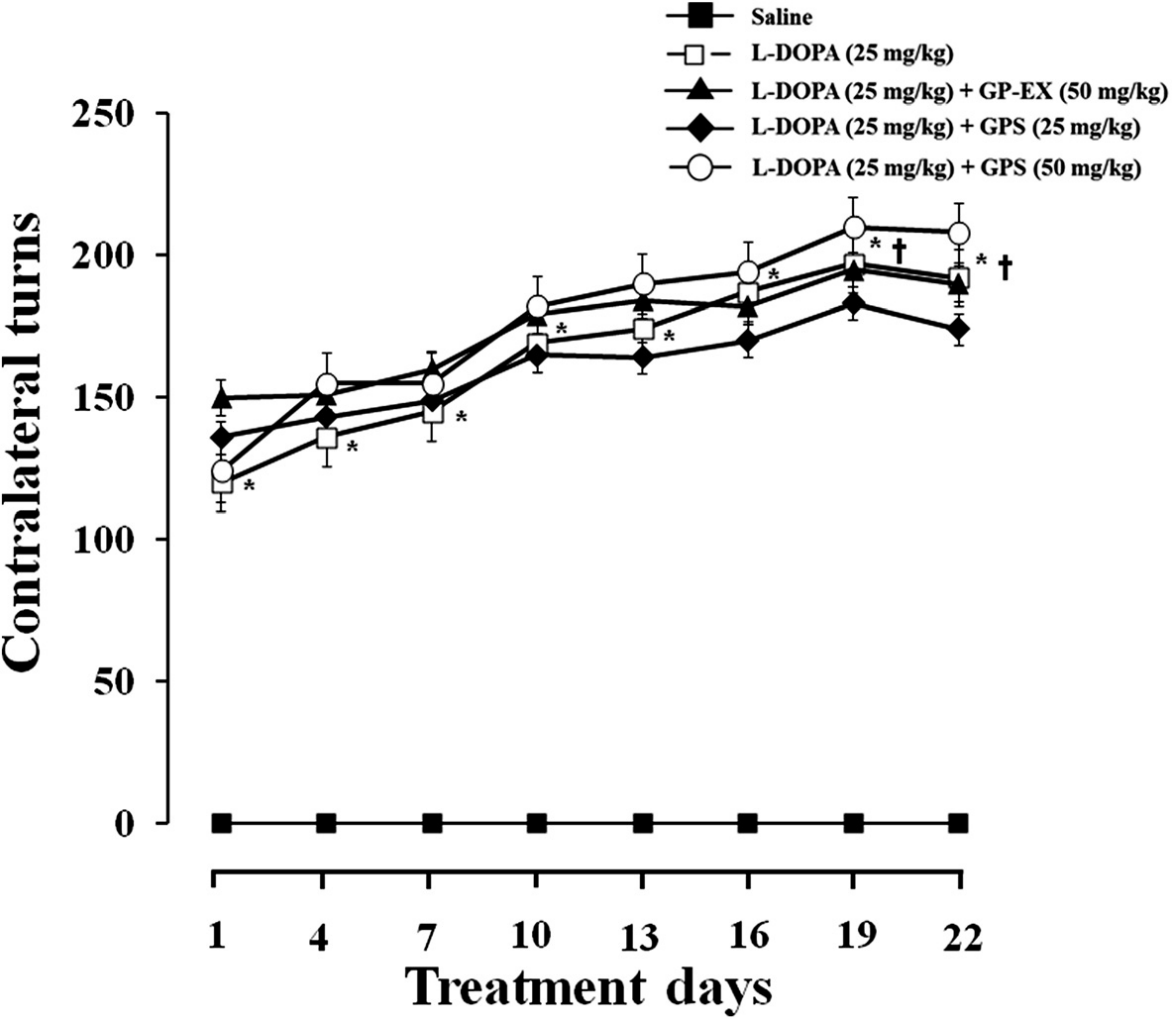
Effects of GPS and GP-EX on contralateral rotation behavior in 6-OHDA-lesioned rats. L-DOPA (25 mg/kg, i.p.) and benserazide (15 mg/kg, i.p.) treatment was started 6 weeks after the 6-OHDA-lesion once a day for 22 days. GPS (25 and 50 mg/kg, p.o.) and GP-EX (50 mg/kg, p.o.) were given 30 min prior to L-DOPA treatment once a day for 22 days. The contralateral rotation behavior was monitored for 1 h on the indicated day. The results are expressed as mean ± S.E.M. for 8–10 animals/group. –■–, saline treatment; −□–, L-DOPA; −▲–, L-DOPA + GP-EX (50 mg/kg); −◆–, L-DOPA + GPS (25 mg/kg); −○–, L-DOPA + GPS (50 mg/kg). ^*^ *P* < 0.05 compared with saline-treated group at each treatment day (non-parametric Kruskal-Wallis one-way ANOVA test); ^†^ *P* < 0.05 compared with the score of day-1 (Friedman repeated measures ANOVA test).

GPS (25 and 50 mg/kg) and GP-EX (50 mg/kg) were co-treated with L-DOPA administration of 6-OHDA-lesioned rats to determine their effects on rotational behavior. L-DOPA administration (25 mg/kg) in 6-OHDA-lesioned rats produced a significant increase in contralateral turns, compared with saline-treated 6-OHDA-lesioned group (Figure 4). Treatment with GPS (25 and 50 mg/kg) or GP-EX (50 mg/kg) for 22 days in 6-OHDA-lesioned rats treated with L-DOPA also slightly increased the number of contralateral turns, compared with 6-OHDA-lesioned rats treated with L-DOPA, but it was not significant (Figure 4). In addition, a slight enhancement of contralateral rotations on the anti-parkinsonian effects of L-DOPA was observed in GPS (50 mg/kg)-treated group (Figure 4). However, no significant interaction between L-DOPA and GPS or GP-EX was observed (Figure 4).

### Effects of GPS and GP-EX on ΔFosB expression

**Figure 5:**
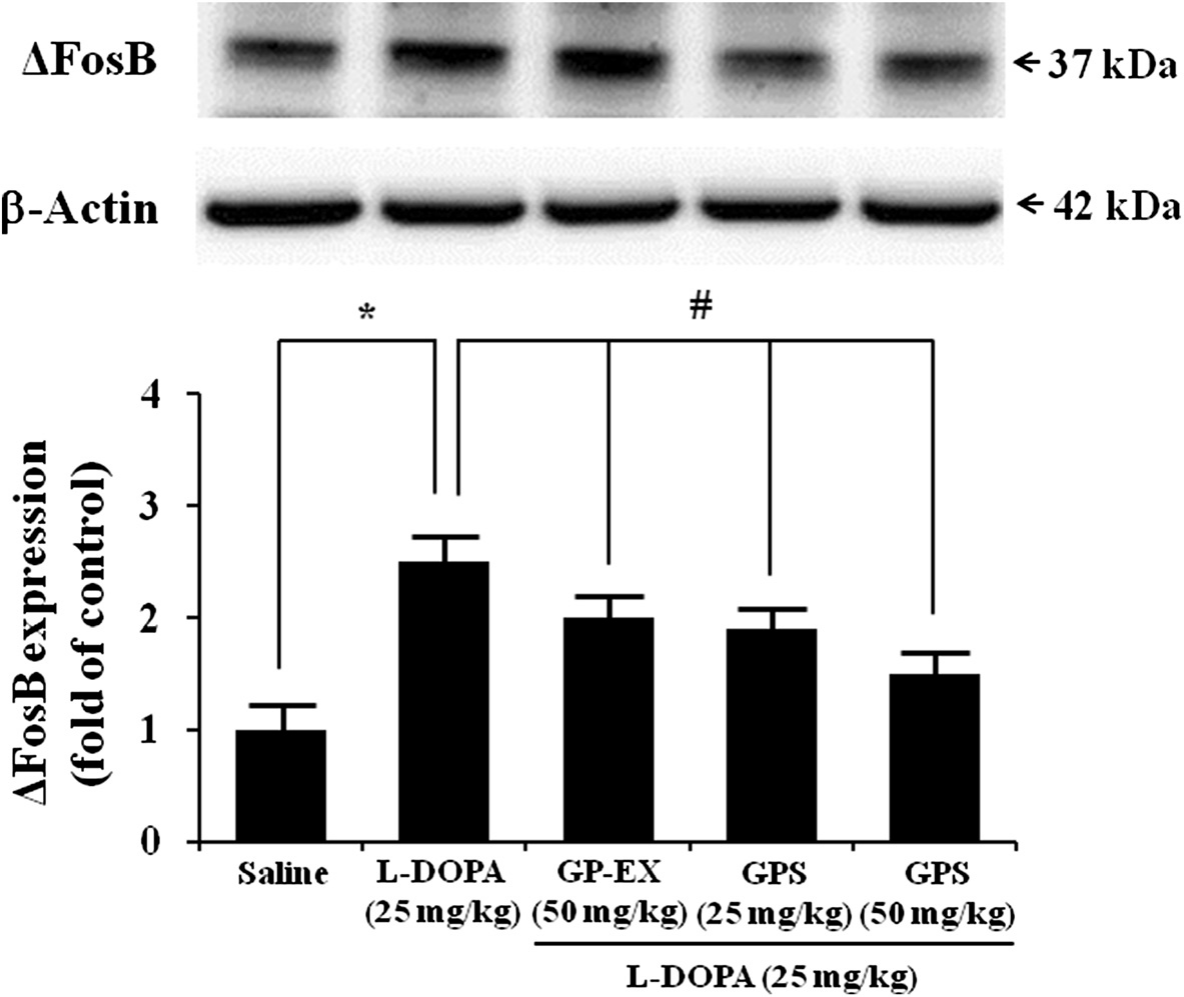
Effects of GPS and GP-EX on L-DOPA-induced expression of ∆FosB in 6-OHDA-lesioned rats. ∆FosB expression was evaluated by western blotting of proteins extracted from the 6-OHDA-lesioned striatum 1 h after the final L-DOPA treatment. Immunoblot images were detected by antibodies against ∆FosB and β-actin using western blotting analysis. Values of the relative density ratios of ∆FosB expression, which are normalized by β-actin, are expressed in arbitrary units as compared with saline-treated group or L-DOPA alone-treated group. The position of molecular size markers is indicated as kDa. The results are expressed as mean ± S.E.M. for 8–10 animals/group. ^*^ *P* < 0.05 compared with saline-treated group; ^#^ *P* < 0.05 compared with L-DOPA alone-treated group (one-way ANOVA followed by Tukey’s test).

Degeneration of dopaminergic neurons by both 6-OHDA and L-DOPA treatment induced an overall increase in striatal levels of ∆FosB, which was measured by western blot (Figure 5). L-DOPA (25 mg/kg) administration resulted in a 2.5-fold (*P* < 0.05) increase in expression of ∆FosB in 6-OHDA-lesioned rats, compared with 6-OHDA-lesioned rats treated with saline (Figure 5). However, co-treatment with GPS (25 and 50 mg/kg) prior to L-DOPA administration decreased the expression of ∆FosB to 1.9- and 1.5-fold (*P* < 0.05), compared with 6-OHDA-lesioned rats treated with L-DOPA (Figure 5). Co-treatment with GP-EX (50 mg/kg) prior to L-DOPA administration also decreased the expression of ∆FosB (2.0-fold, *P* < 0.05), compared with 6-OHDA-lesioned rats treated with L-DOPA (Figure 5).

### Effects of GPS and GP-EX on ERK1/2 phosphorylation

**Figure 6:**
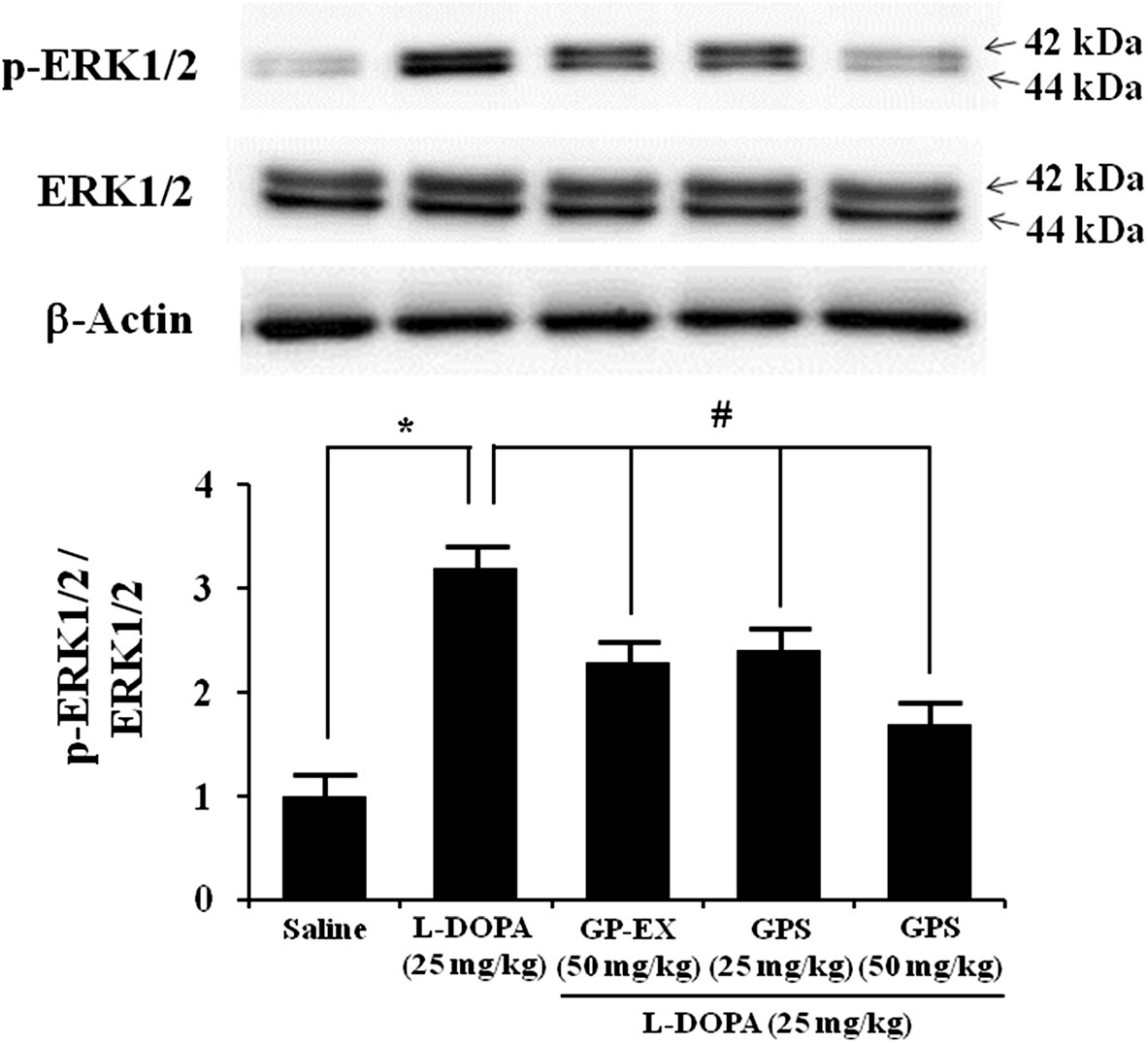
Effects of GPS and GP-EX on L-DOPA-induced phosphorylation of ERK1/2 in 6-OHDA-lesioned rats. ERK1/2 phosphorylation (p-ERK1/2) was evaluated by western blotting of proteins extracted from the 6-OHDA-lesioned striatum 1 h after the final L-DOPA treatment. Immunoblot images were detected by antibodies against phospho-ERK1/2, ERK1/2 and β-actin using western blotting analysis. Values of the relative density ratios of p-ERK1/2/ERK1/2 are normalized and expressed in arbitrary units as compared with saline-treated group or L-DOPA alone-treated group. The position of molecular size markers is indicated as kDa. The results are expressed as mean ± S.E.M. for 8–10 animals/group. *P < 0.05 compared with saline-treated group; ^#^ *P* < 0.05 compared with L-DOPA alone-treated group (one-way ANOVA followed by Tukey’s test).

L-DOPA (25 mg/kg) administration resulted in 3.2-fold (*P* < 0.05) increase in ERK1/2 phosphorylation in 6-OHDA-lesioned rats, compared with 6-OHDA-lesioned rats treated with saline (Figure 6). Co-treatment with GPS (25 and 50 mg/kg) prior to L-DOPA administration (25 mg/kg) reduced ERK1/2 phosphorylation (2.4-fold and 1.7-fold, *P* < 0.05) respectively, compared with 6-OHDA-lesioned rats treated with L-DOPA (Figure 6). Co-treatment with GP-EX (50 mg/kg) prior to L-DOPA administration (25 mg/kg) also decreased ERK1/2 phosphorylation (2.3-fold, *P* < 0.05), compared with 6-OHDA-lesioned rats treated with L-DOPA (Figure 6).

## Discussion

GPS and GP-EX showed anxiolytic effects on affective disorders in MPTP-lesioned mouse model of PD with or without L-DOPA administration [13]. GPS (100–200 mg/kg) showed protective effects in MPTP-lesioned mouse model of PD [14]. GP-EX also has protective effects on neurotoxicity by modulating TH neuronal cell death and dopamine levels in 6-OHDA-lesioned rat model of PD [15]. On the basis of these results, we investigated the effects of GPS and GP-EX on the developments of LID in 6-OHDA-lesioned rat model of PD.

The body AIMs score such as axial, limb and orolingual AIMs score is increased in LID in 6-OHDA-lesioned animal model of PD [17]. However, the locomotive AIMs score does not necessarily provide a behavioral correlate of LID [18]. In this study, LID expression was significantly increased by chronic L-DOPA administration for 22 days in 6-OHDA-lesioned rats (Figure 2). However, co-treatment with GPS (25 and 50 mg/kg) or GP-EX (50 mg/kg) prior to L-DOPA administration significantly attenuated the development of LID as determined by body AIMs score (Figures 2 and 3A–C). In contrast, the locomotive AIMs score was not altered by co-treatment with GPS (25 and 50 mg/kg) or GP-EX (50 mg/kg) prior to L-DOPA administration (Figure 3).

L-DOPA treatment increased the number of contralateral rotations in 6-OHDA-lesioned rats, indicating behavior sensitization. The contralateral rotations are indicative of anti-parkinsonian efficacy. However, it is also suggested that the rotational behaviors are a dyskinesia symptom [17]. In this study, treatment with GPS (25 and 50 mg/kg) or GP-EX (50 mg/kg) in 6-OHDA-lesioned rats administered with L-DOPA slightly increased the contralateral rotations, but it was not significant (Figure 4), suggesting that GPS (25 and 50 mg/kg) and GP-EX (50 mg/kg) treatment did not produce a significant behavior sensitization. Furthermore, these results indicate that the anti-dyskinetic efficacy of GPS and GP-EX is not due to an attenuation of L-DOPA efficacy.

The striatal ∆FosB is induced in the development of LID in PD rat models [9]. 6-OHDA-lesioned rats and L-DOPA administration in saline-lesioned rats do not affect ∆FosB [19,20]. In addition, the phosphorylated levels of ERK1/2 are not affected by saline treatment in 6-OHDA-lesioned rats [21]. The pronounced phosphorylation of ERK1/2 in the striatum of 6-OHDA-lesioned rats provides a molecular counterpart to the induction of AIMs by L-DOPA administration [21]. The present study showed the significant ∆FosB expression and ERK1/2 phosphorylation after L-DOPA administration in the striatum of 6-OHDA-lesioned rats (Figures 5 and 6). However, the increased ∆FosB expression after L-DOPA administration in the striatum of 6-OHDA-lesioned rats was reduced by treatment with either GPS (25 and 50 mg/kg) or GP-EX (50 mg/kg) prior to L-DOPA administration (Figure 5). GPS (25 and 50 mg/kg) and GP-EX (50 mg/kg) also decreased ERK1/2 phosphorylation in 6-OHDA-lesioned rats after L-DOPA administration. These results suggest that the effects of GPS and GP-EX on the development of LID are mediated by the modulation of ∆FosB expression and ERK1/2 phosphorylation in 6-OHDA-lesioned rats.

It is reported that LID affects up to 80% of PD patients after L-DOPA administration for 5–10 years and some of PD patients have to stop the therapy due to severe LID [22], which is difficult to treat. The pathogenesis of LID remains incompletely understood presently. However, it is known that dyskinesias appear only after dopaminergic therapy and the presence of dopaminergic cell loss in the substantia nigra [23].

Furthermore, there is evidence that supersensitivity in dopamine-depleted striatum results from pulsatile stimulation of postsynaptic dopamine receptor [24]. The increases in ∆FosB expression after L-DOPA administration have been shown to be associated with the supersensitive responses to L-DOPA induced by dopaminergic stimulation in dopamine depleted striatum [8]. The sustained activation of ERK1/2 by L-DOPA administration also reflects the supersensitivity of dopamine receptor-dependent signaling in dopamine-depleted striatum [21]. In addition, L-DOPA induces ∆FosB expression in striatal neurons by involving D_1_ receptors and ERK1/2 phosphorylation [8,10]. In 6-OHDA-lesioned striatum, both D_1_ and D_2_ receptor agonists have been reported to significantly induce ERK1/2 activation [10,11]. These results suggest that changes in ∆FosB expression and ERK signaling system are associated with the supersensitive responses to dopaminergic stimulation, which is implicated in LID in dopamine-depleted striatum. Recently, the sustained ERK1/2 phosphorylation induced by L-DOPA has been shown to possibly lead to dopaminergic neuronal cell death in PC12 cells [25], and GPS has shown protective effects on 1-methyl-4-phenylpyridinium (MPP^+^)-induced oxidative injury of dopaminergic neurons in primary culture [26]. Therefore, the protective effects of GPS and GP-EX on dopaminergic neuronal cells can be involved in the inhibition of the development of LID.

GPS is a potent free radical scavenger, which strongly increases the superoxide dismutase activity [27]. GPS shows protective effects in aortic endothelial cells against oxidative damage [28] and on oxidative stress induced by glutamate-induced neurotoxicity [29]. GP-EX also has been reported to have anti-stress and immunomodulatory effects in mice [16,30]. GP-EX has a protective function against chronic stress by modulation of c-Fos expression [16]. Furthermore, L-DOPA increases nitric oxide production in the striatum which is associated with PD [31]. Neuronal nitric oxide synthase inhibition attenuates the expression of LID [32]. Daily repeated L-DOPA administration also increases the nitric oxide generation through activation of neural nitric oxide synthase and this nitric oxide develops LID through a post-synaptic mechanism by the accumulation of ∆FosB [33]. In contrast, it is suggested that the development of LID is closely associated with progression of pre-synaptic dopaminergic neurodegeneration in the substantia nigra [34]. It is therefore suggested that the ameliorating effects of GPS and GP-EX on LID might be mediated by scavenging the ROS formation in the striatum of 6-OHDA-lesioned rats. However, whether the functions of GPS and GP-EX on reducing ΔFosB levels are involved in the post-synaptic mechanism needs to be studied further.

The dose of GPS (50–200 mg/kg) and GP-EX (50–400 mg/kg) treatments does not exhibit adverse effects, such as weight loss, diarrhea, vomiting, and death [16]. The LD_50_ values of total GPS are 755–838 mg/kg (injected into the abdominal cavity) and 402 (±18.2 mg/kg, i.p.) in mice [35], indicating that GPS and GP-EX are low toxicity therapeutic agents.

## Conclusion

The present study demonstrated that GPS (25 and 50 mg/kg) and GP-EX (50 mg/kg) not only attenuated the development of LID, but also reduced both ∆FosB expression and ERK1/2 phosphorylation in 6-OHDA-lesioned rats treated with chronic L-DOPA. GPS and GP-EX also had no negative effects on the anti-parkinsonian efficacy of L-DOPA, which was defined by the contralateral rotational tests. It is therefore proposed that GPS and GP-EX as very low toxic agents can be helpful in preventing the L-DOPA-induced adverse or toxic effects for PD patients, as well as in slowing down the progression of LID. The clinical applications need to be studied further.

## Methods

### Materials

GPS was purchased from Ankang Dongke Maidisen Nature Pharmaceutical Co. (purity > 99%, confirmed by HPLC analysis) (Xi’an, China) [14,29]. GP was obtained from the Wonkwang Food Manufacturing Co. (Geochang, Korea) and a voucher specimen of the herbal leaves of GP was deposited at the herbarium of the College of Pharmacy, Chungbuk National University (Cheongju, Korea). The air-dried leaves of GP (1 kg) were extracted with ethanol (80%, v/v) and the ethanol extracts were evaporated to dryness under reduced pressure and temperature (GP-EX, 97.2 g, yield, 9.7%, w/w).

L-DOPA, 6-OHDA, benserazide hydrochloride, and apomorphine were purchased from Sigma-Aldrich (St. Louis, MO, USA). Rabbit polyclonal primary antibodies and anti-rabbit IgG HRP-linked secondary antibodies against ERK1/2, phospho-ERK1/2, ∆FosB and β-actin were purchased from Cell Signaling Technology Inc. (Beverly, MA, USA). All other chemicals were of analytical grade.

### Experimental animals

Rats (Sprague–Dawley, male, 200–250 g) were purchased from Samtako (Osan, Korea) and housed under standard conditions of temperature (23 ± 2°C), humidity (60 ± 5%), and illumination (12-h light–dark cycle lighted on at 07:00) with ad libitum access to standard rat food and water. All experimental procedures were approved by the guidelines of Animal Ethics Committee of Chungbuk National University Laboratory Animal Research Center (Approval no. CBNUA-708-141-01) and were conducted according to the National Institutes of Health (NIH) guidelines.

### Unilateral 6-OHDA lesion

Unilateral 6-OHDA lesions were conducted as described previously [11,17]. The rats were anesthetized intraperitoneally with Zoletil 50 (100 mg/kg, Virbac, Carros, France) and placed in a stereotaxic stand (David Kopf Instruments, Tujunga, CA, USA). The coordinates for the medial forebrain bundle were measured accurately (antero-posterior, AP: −2.5 mm; medio-lateral, ML: +2.0 mm; dorso-ventral, DV: −8.5 mm; relative to bregma). Next, 6-OHDA (8 μg/2 μl in saline solution containing 0.05% of L-ascorbic acid) was single injected into the left medial forebrain bundle at 1 μl/min using a Hamilton syringe. After the injection, the needle was left in place for 5 min before being retracted in order to allow for complete diffusion of the medium. The rats were left in the stand until they had recovered from the anesthesia. In order to assess the efficacy of the lesion, all rats were tested for apomorphine (0.5 mg/kg, s.c.)-induced rotation at 3 weeks after the 6-OHDA lesions. Rats showing more than 150 rotations/30 min were selected for this study [36]. In these states, the striatal levels of dopamine in 6-OHDA-lesioned rats decreased to 40.9–47.1% as compared with control group (dopamine levels of control group, 7.12 ± 0.75 ng/mg tissue).

### Experimental design

The experimental rats were randomly divided into five groups (n = 8–10 per group) 6 weeks after the 6-OHDA lesion [17]. The 6-OHDA-lesioned groups were treated with saline (0.9%, i.p.) or both L-DOPA (25 mg/kg, i.p.) and benserazide (15 mg/kg, i.p.) at 10 am once a day for 22 days. In addition, the 6-OHDA-lesioned groups treated with L-DOPA (25 mg/kg, i.p.) were treated with either GPS (25 and 50 mg/kg) or GP-EX (50 mg/kg) orally (p.o.) 30 min prior to L-DOPA administration once a day for 22 days [36] (Figure 1). After the last day of GPS, GP-EX and L-DOPA treatment, rats were tested for last AIMs score and contralateral rotation. Finally, the rats were sacrificed for biochemical analysis including ∆FosB expression and ERK1/2 phosphorylation.

### Behavioral measurements

Rats were monitored for AIMs according to previously published procedures and methods [17,37]. After treatment with L-DOPA, rats were observed individually for 1 min every 20 min for 180 min period following dose of L-DOPA. Observation was performed by trained observers who were blinded to the animal groupings and experimental conditions. The AIMs were scored for exhibition of the following four categories: (1) axial AIMs, twisting movement of the neck, trunk and head toward the side contralateral to the 6-OHDA lesion; (2) limb AIMs, repetitive jerky movements or dystonic posturing of the forelimb contralateral to the 6-OHDA lesion; (3) orolingual AIMs, purposeless jaw movements and contralateral tongue protrusion without the presence of food or other objects; (4) locomotive AIMs, increased circular locomotion with contralateral side bias. During the 1 min observation period, the four subtypes were scored on a scale from 0 to 4 in each rat based on the following criteria: 0, not present; 1, present for less than half of the observation time; 2, present for more than half of the observation time; 3, present all the time but suppressible by threatening stimuli; 4, present all the time and not suppressible. The axial, limb, and orolingual AIMs were calculated by adding each of the individual dyskinesia scores as body AIMs (total AIMs). The body AIMs were also expressed by the area under the curve of the each AIMs parameter. Locomotive AIMs rating took into account the rat’s circular movements on a flat floor using its all four limbs, which were counted by only complete 360° turns. The contralateral rotation was counted for 1 h and started at 20 min after L-DOPA [36].

### Western blotting

The rats were deeply anesthetized with Zoletil 50 (100 mg/kg, Virbac, Carros, France) and sacrificed by rapid decapitation 1 h after L-DOPA treatment. The brains were quickly removed and the striatum was isolated on ice and homogenized in a lysis buffer. Protein extracts from the striata of PD rats were prepared from the left striata, and 20 μg protein from each rat was used for western blotting [38]. The primary antibodies used were rabbit polyclonal primary antibody (1:1000 dilution) against ∆FosB, phospho-ERK1/2, ERK1/2 and β-actin. Proteins in samples (20μg) were separated using 10–15% sodium dodecyl sulfate-poly acrylamide gel electrophoresis. Proteins were transferred to polyvinylidene difluoride membrane at 300 mA for 1 h. The blots were blocked for 1 h at room temperature in a fresh blocking buffer (TBS-T containing 5% bovine serum albumin [BSA]) and then incubated overnight at 4°C using primary antibodies diluted 1:1000 in TBS-T with 5% BSA, and for 1 h at room temperature using secondary antibodies (anti-rabbit IgG HRP-linked antibodies, 1:5000 dilution in TBS-T with 5% BSA), according to a standard procedure. The blots were then washed, and the transferred proteins were incubated with ECL substrate solution (Amersham Pharmacia Biotech, Inc., Piscataway, NJ) for 5 min, according to the manufacturer’s instructions, and visualized with a radiographic film.

### Statistical analysis

Protein amounts were determined by a bicinchoninic acid protein assay kit using BSA (Pierce Protein Research Products, Rockford, IL). Behavioral data and group comparisons of dyskinesia intensity scores were analyzed by non-parametric Kruskal-Wallis one-way ANOVA test for multi-group comparisons at each day and Friedman repeated measures ANOVA test for two-group comparisons unless otherwise indicated. Biochemical data were also analyzed by one-way ANOVA followed by Tukey’s test. All data were expressed as mean ± S.E.M. with *P* values of < 0.05 being considered statistically significant.

## Abbreviations

6-OHDA: 6-hydroxydopamine
AIMs: Abnormal involuntary movements
BSA: Bovine serum albumin
ERK1/2: Extracellular signal-regulated kinases
ΔFosB: FBJ murine osteosarcoma viral oncogene homolog B delta
GPS: Gypenosides
GP-EX: Ethanol extract of *Gynostemma pentaphyllum*
L-DOPA: L-3,4-dihydroxyphenylalanine
LID: L-DOPA-induced-dyskinesia
MPP^+^: 1-methyl-4-phenylpyridinium
MPTP: 1-methyl-4-phenyl-1,2,3,6-tetrahydropyridine
PD: Parkinson’s disease
